# Plucky

**Published:** 1889-07

**Authors:** 


PLUCKY.—A doctor of Staten Island, aged 80, is said to have recently married a girl of 16 years. Such an act always seems foolish in that other old fellow.

